# Disodium zinc bis­(sulfate) tetra­hydrate (zinc astrakanite) revisited

**DOI:** 10.1107/S1600536808009719

**Published:** 2008-05-07

**Authors:** M. Enriqueta Díaz de Vivar, Sergio Baggio, Andrés Ibáñez, Ricardo Baggio

**Affiliations:** a Universidad Nacional de la Patagonia, Sede Puerto Madryn, and CenPat, CONICET, 9120 Puerto Madryn, Chubut, Argentina; b Departamento de Física, Facultad de Ciencias Físicas y Matemáticas, Universidad de Chile, and CIMAT, Casilla 487-3, Santiago de Chile, Chile; c Departamento de Física, Comisión Nacional de Energía Atómica, Buenos Aires, Argentina

## Abstract

We present a new low-temperature refinement of disodium zinc bis­(sulfate) tetra­hydrate {systematic name: poly[tetra-μ-aqua-di-μ-sulfato-zinc(II)disodium(I)]}, [Na_2_Zn(SO_4_)_2_(H_2_O)_4_]_*n*_ or Zn astrakanite, which is an upgrade of previously reported data [Bukin & Nozik (1974 ▶). *Zh. Strukt. Khim.* **15**, 712–716]. The compound is part of an isostructural family containing the Mg (the original astrakanite mineral), Co and Ni species. The very regular ZnO(aqua)_4_O(sulfate)_2_ octa­hedra lie on centres of symmetry, while the rather distorted NaO(aqua)_2_O(sulfate)_4_ octa­hedra appear at general positions, linked into a three-dimensional network by the bridging water mol­ecules and the fully coordinated sulfate groups.

## Related literature

For related literature, see: Rumanova (1958 ▶); Giglio (1958 ▶); Bukin & Nozik (1974 ▶, 1975 ▶); Díaz de Vivar *et al.* (2006 ▶).
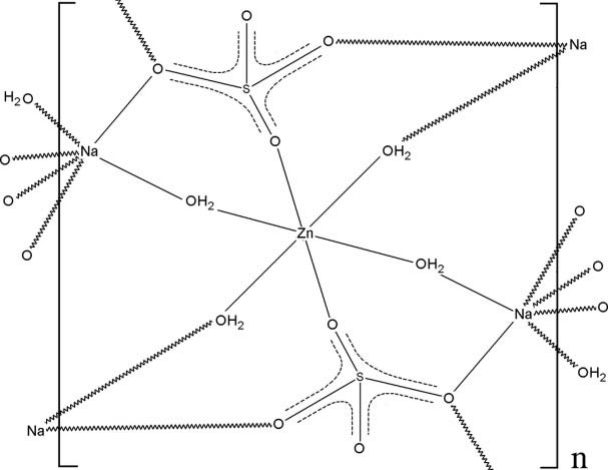


## Experimental

### 

#### Crystal data

[Na_2_Zn(SO_4_)_2_(H_2_O)_4_]
*M* *_r_* = 375.53
Monoclinic, 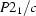

*a* = 5.5075 (2) Å
*b* = 8.2127 (3) Å
*c* = 11.0559 (4) Å
β = 99.958 (10)°
*V* = 492.54 (3) Å^3^
*Z* = 2
Mo *K*α radiation
μ = 3.07 mm^−1^
*T* = 170 (2) K
0.30 × 0.20 × 0.10 mm

#### Data collection

Bruker SMART CCD diffractometer
Absorption correction: multi-scan (*SADABS*; Sheldrick, 1996 ▶) *T*_min_ = 0.452, *T*_max_ = 0.728
3533 measured reflections
1080 independent reflections
1062 reflections with *I* > 2σ(*I*)
*R*_int_ = 0.012

#### Refinement

*R*[*F* ^2^ > 2σ(*F* ^2^)] = 0.017
*wR*(*F* ^2^) = 0.054
*S* = 1.00
1080 reflections
96 parameters
6 restraints
H atoms treated by a mixture of independent and constrained refinement
Δρ_max_ = 0.29 e Å^−3^
Δρ_min_ = −0.53 e Å^−3^

### 

Data collection: *SMART* (Bruker, 2001 ▶); cell refinement: *SAINT* (Bruker, 2001 ▶); data reduction: *SAINT*; program(s) used to solve structure: *SHELXS97* (Sheldrick, 2008 ▶); program(s) used to refine structure: *SHELXL97* (Sheldrick, 2008 ▶); molecular graphics: *SHELXTL* (Sheldrick, 2008 ▶); software used to prepare material for publication: *SHELXTL* and *PLATON* (Spek, 2003 ▶).

## Supplementary Material

Crystal structure: contains datablocks global, I. DOI: 10.1107/S1600536808009719/fi2061sup1.cif

Structure factors: contains datablocks I. DOI: 10.1107/S1600536808009719/fi2061Isup2.hkl

Additional supplementary materials:  crystallographic information; 3D view; checkCIF report

## Figures and Tables

**Table 1:** Selected bond lengths (Å)

Zn1—O1*W*	2.0636 (11)
Zn1—O3	2.0952 (11)
Zn1—O2*W*	2.1285 (11)
Na1—O2^i^	2.3603 (12)
Na1—O4^ii^	2.3786 (12)
Na1—O1	2.4016 (12)
Na1—O1*W*	2.4017 (12)
Na1—O2^iii^	2.4224 (13)
Na1—O2*W*^iv^	2.5694 (13)

Symmetry codes: (i) 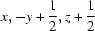; (ii) 
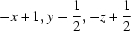
; (iii) 
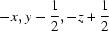
; (iv) 
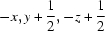
.

**Table 2:** Hydrogen-bond geometry (Å, °)

*D*—H⋯*A*	*D*—H	H⋯*A*	*D*⋯*A*	*D*—H⋯*A*
O1*W*—H1*WA*⋯O1^iii^	0.800 (17)	1.916 (17)	2.6977 (17)	165 (3)
O1*W*—H1*WB*⋯O4^v^	0.832 (16)	1.901 (16)	2.7288 (17)	173 (2)
O2*W*—H2*WA*⋯O1^ii^	0.826 (16)	2.051 (18)	2.8468 (16)	162 (2)
O2*W*—H2*WB*⋯O4^vi^	0.805 (16)	2.15 (2)	2.8779 (16)	151 (3)

Symmetry codes: (ii) 
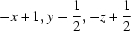
; (iii) 
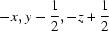
; (v) 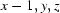; (vi) 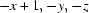.

**Fig. 1.**
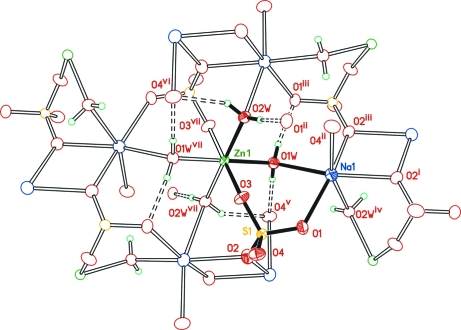
A (100) view of the structure with the independent atoms drawn in full 50% displacemenet ellipsoids and full bonds. The symmetry related part, in open ellipsoids and hollow bonds. Hydrogen interactions drawn in broken lines. Symmetry codes: (i) x, -y + 1/2, z + 1/2; (ii) -x + 1, y - 1/2, -z + 1/2; (iii) -x, y - 1/2, -z + 1/2; (iv) -x, y + 1/2, -z + 1/2. (v) x - 1, y, z; (vi) -x + 1, -y, -z; (vii) -x, -y, -z.

**Fig. 2.**
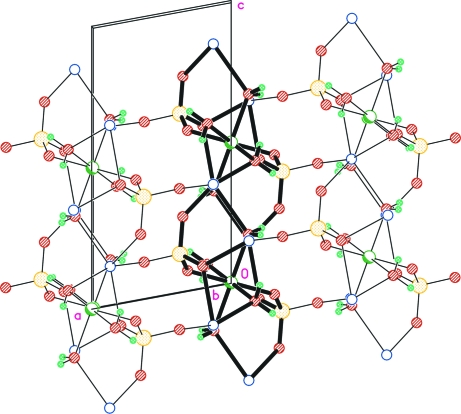
Packing view down the <010> direction showing a side view of the planar structures, and the way they interact through the O4—Na1 bonds along [100]. H-bonds omited in this view, for clarity.

**Fig. 3.**
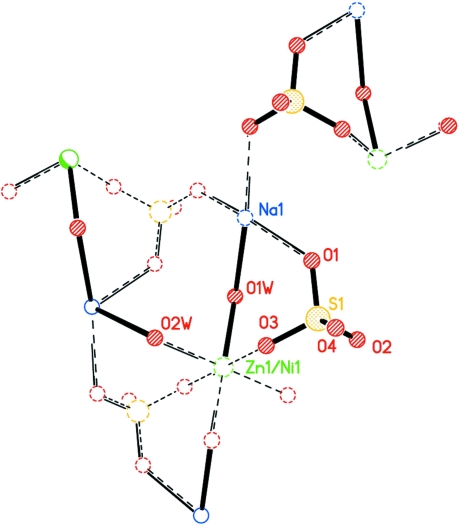
Least squares fit of an extended atomic group in (I), in full lining, and its Ni counterpart (Díaz de Vivar et al., 2006), in dashed lining. Note the almost perfect overlap of both structures.

## supplementary crystallographic information

### Comment

The original mineral astrakanite [Na_2_M(SO_4_)_2_(H_2_O)_4_], *M* = Mg, structurally characterized almost 50 years ago (Rumanova, 1958), gave its name to a whole isostructural family, of which some members have been known for a long while (*M* = Zn, Giglio, 1958; Bukin & Nozik, 1974; *M* = Co, Bukin & Nozik, 1975), while the Ni analogue has been only recently reported, (Díaz de Vivar *et al.*, 2006). We present herein an improved, low temperature data refinement of the zinc member of the group, Na_2_Zn(SO_4_)_2_(H_2_O)_4_ (I), unwittingly obtained as a byproduct while looking for something else (See experimental section).

Fig. 1 shows the asymmetric unit of (I) as well as part of its close environment, and Table 1 presents some selected bond distances. The structure consists of ZnO(aqua)_4_O(sulf)_2_ and NaO(aqua)_2_O(sulf)_4_ octahedra in a 1:2 ratio, linked through two bridging water molecules (O1W, O2W) and the fully coordinated sulfato groups.

Zn cations lay on centers of symmetry and their coordination polyhedra defined by O3, O1w, O2w and their respective centrosymmetric counterparts are quite regular, possibly due to the large number of geometrically unconstrained aqua molecules (Parameters range: Zn—O,2.0636 (11)–2.1285 (11) Å; (O—Zn—O)*_cis_*, 87.38 (5)–92.62 (5)°; (O—Zn—O)*_trans_*, 180.°, fixed by symmetry). Na cations, instead, occupy general positions and, contrasting the former, their O(sulf)-rich coordination octahedra appear as quite irregular (Parameters range: Na—O,2.3603 (12)–2.5694 (13) Å; (O—Na—O)*_cis_*, 74.93 (4)–112.94 (4)°; (O—Na—O)*_trans_*, 155.87 (5)–162.11 (5)°).

The geometry of the sulfate anion is rather regular, with S—O distances in the range 1.4619 (11) to 1.4878 (11) Å and angles from 107.38 (5) to 110.89 (7)°. The group exhibits a complex µ_5_-κ^4^-O:*O*':*O*'':*O*''' coordination, binding in a monocoordinated fashion to Zn through O3 and to Na through O1 and O4, while bridging two Na cations through O2. The result of this intricate interconnectivity is the formation of broad two-dimensional structures parallel to (100) containing both types of polyhedra (Fig.1) and internally linked by the two bridging aqua and O atoms O1, O2 and O3 from the sulfate anion.

These "planes", in turn, are interconnected along a single "strong" interaction, the O4—Na1 bonds between sulfate O4 atoms from a given layer and Na1 cations from their neighbours (Fig. 2).

Also H-bonding interactions (Table 2) contribute to the intraplane (*via* O1W, entries 1 and 2) and interplane (*via* O2W, entries 3 and 4) cohesion.

It is worth noting that O1 and O4 act as the only (double) acceptors for H-bonding. In analyzing the S—O bond lengths, it appears that S1—O1 and S1—O4 present precisely the longest distances suggesting a slight weakening effect on the S—O covalent link due to the oxygen involvement in H interactions.

Even though the isostructural character of (I) with the rest of the strakanite family is obvious by inspection, the low precision with which the Mn and Co members have been reported leaves comparison with the Ni moiety as the only relevant one. In this respect, both structures are almost undistinguishable, as proved by the least squares fit of the extended group shown in Fig. 3, where the maximum departure amounts for less than 0.05Å for atom O2W.

### Experimental

The compound was obtained an an unintended product in a synthesis of Zn(II) complexes. Recently prepared anysaldehyde bisulfitic derivative (60 mg) were dissolved in 5 ml of water and mixed with an aqueous solution of Zn acetate (112 mg/5 ml). The aqueous mixture was left in a methanol atmosphere, until colourless cubic crystals were obtained.

### Refinement

Hydrogen atoms pertaining to water molecules were found in the difference- Fourier synthesis and refined with restrained O—H:0.82 (2) Å, H···H:1.35 (2) Å, but free isotropic displacement parameters.

### Crystal data

**Table:**

[Na_2_Zn(SO_4_)_2_(H_2_O)_4_]	*F*_000_ = 376
*M**_r_* = 375.53	*D*_x_ = 2.539 Mg m^−^^3^
Monoclinic, *P*2_1_/*c*	Mo *K*α radiation λ = 0.71073 Å
Hall symbol: -P 2ybc	Cell parameters from 3942 reflections
*a* = 5.5075 (2) Å	θ = 3.8–26.7º
*b* = 8.2127 (3) Å	µ = 3.07 mm^−^^1^
*c* = 11.0559 (4) Å	*T* = 170 (2) K
β = 99.9580 (10)º	Prism, colourless
*V* = 492.54 (3) Å^3^	0.30 × 0.20 × 0.10 mm
*Z* = 2	

### Data collection

**Table:**

Bruker SMART CCD diffractometer	1080 independent reflections
Radiation source: fine-focus sealed tube	1062 reflections with *I* > 2σ(*I*)
Monochromator: graphite	*R*_int_ = 0.012
*T* = 170(2) K	θ_max_ = 27.9º
φ and ω scans	θ_min_ = 3.1º
Absorption correction: multi-scan(SADABS; Sheldrick, 1996)	*h* = −6→7
*T*_min_ = 0.452, *T*_max_ = 0.728	*k* = −10→10
3533 measured reflections	*l* = −13→14

### Refinement

**Table:**

Refinement on *F*^2^	Secondary atom site location: difference Fourier map
Least-squares matrix: full	Hydrogen site location: inferred from neighbouring sites
*R*[*F*^2^ > 2σ(*F*^2^)] = 0.017	H atoms treated by a mixture of independent and constrained refinement
*wR*(*F*^2^) = 0.054	*w* = 1/[σ^2^(*F*_o_^2^) + (0.0408*P*)^2^ + 0.265*P*] where *P* = (*F*_o_^2^ + 2*F*_c_^2^)/3
*S* = 1.00	(Δ/σ)_max_ < 0.001
1080 reflections	Δρ_max_ = 0.29 e Å^−^^3^
96 parameters	Δρ_min_ = −0.53 e Å^−^^3^
6 restraints	Extinction correction: SHELXL97 (Sheldrick, 2008), Fc^*^=kFc[1+0.001xFc^2^λ^3^/sin(2θ)]^-1/4^
Primary atom site location: structure-invariant direct methods	Extinction coefficient: 0.080 (4)

### Special details

**Table:**

Geometry. All e.s.d.'s (except the e.s.d. in the dihedral angle between two l.s. planes) are estimated using the full covariance matrix. The cell e.s.d.'s are taken into account individually in the estimation of e.s.d.'s in distances, angles and torsion angles; correlations between e.s.d.'s in cell parameters are only used when they are defined by crystal symmetry. An approximate (isotropic) treatment of cell e.s.d.'s is used for estimating e.s.d.'s involving l.s. planes.

### Fractional atomic coordinates and isotropic or equivalent isotropic displacement parameters (Å^2^)

**Table:**

	*x*	*y*	*z*	*U*_iso_*/*U*_eq_	
Zn1	0.0000	0.0000	0.0000	0.00887 (13)	
Na1	0.12607 (11)	0.07173 (8)	0.36217 (5)	0.01231 (17)	
S1	0.37405 (6)	0.28842 (4)	0.13609 (3)	0.00821 (14)	
O1	0.3516 (2)	0.27120 (14)	0.26765 (10)	0.0127 (2)	
O2	0.2085 (2)	0.41630 (14)	0.07871 (10)	0.0136 (3)	
O3	0.3186 (2)	0.13129 (14)	0.07174 (10)	0.0134 (2)	
O4	0.63500 (19)	0.32955 (13)	0.13056 (10)	0.0127 (2)	
O1W	−0.1247 (2)	0.03807 (16)	0.16331 (10)	0.0110 (2)	
O2W	0.1753 (2)	−0.21442 (13)	0.08065 (10)	0.0115 (2)	
H1WA	−0.215 (5)	−0.032 (3)	0.179 (2)	0.028 (7)*	
H1WB	−0.207 (4)	0.123 (2)	0.156 (2)	0.023 (6)*	
H2WA	0.299 (4)	−0.203 (3)	0.1341 (18)	0.021 (6)*	
H2WB	0.213 (5)	−0.278 (3)	0.032 (2)	0.034 (7)*	

### Atomic displacement parameters (Å^2^)

**Table:**

	*U*^11^	*U*^22^	*U*^33^	*U*^12^	*U*^13^	*U*^23^
Zn1	0.01021 (18)	0.00753 (17)	0.00909 (17)	−0.00024 (7)	0.00231 (11)	−0.00054 (7)
Na1	0.0133 (3)	0.0116 (3)	0.0118 (3)	−0.0002 (2)	0.0015 (2)	0.0005 (2)
S1	0.0083 (2)	0.0075 (2)	0.0089 (2)	0.00016 (12)	0.00150 (13)	−0.00049 (12)
O1	0.0156 (6)	0.0130 (5)	0.0101 (5)	0.0014 (4)	0.0034 (4)	0.0013 (4)
O2	0.0149 (5)	0.0139 (6)	0.0119 (5)	0.0051 (4)	0.0017 (4)	0.0017 (4)
O3	0.0111 (5)	0.0109 (5)	0.0179 (5)	−0.0016 (4)	0.0022 (4)	−0.0054 (4)
O4	0.0103 (5)	0.0110 (5)	0.0176 (5)	−0.0021 (4)	0.0042 (4)	−0.0016 (4)
O1W	0.0114 (5)	0.0098 (5)	0.0122 (5)	0.0000 (4)	0.0035 (4)	0.0002 (4)
O2W	0.0120 (5)	0.0100 (5)	0.0118 (5)	0.0007 (4)	−0.0002 (4)	−0.0017 (4)

### Geometric parameters (Å, °)

**Table:**

Zn1—O1W^i^	2.0636 (11)	Na1—O2W^v^	2.5694 (13)
Zn1—O1W	2.0636 (11)	Na1—Na1^vi^	3.7507 (12)
Zn1—O3	2.0952 (11)	S1—O2	1.4619 (11)
Zn1—O3^i^	2.0952 (11)	S1—O3	1.4797 (11)
Zn1—O2W	2.1285 (11)	S1—O1	1.4876 (11)
Zn1—O2W^i^	2.1285 (11)	S1—O4	1.4878 (11)
Na1—O2^ii^	2.3603 (12)	O1W—H1WA	0.800 (17)
Na1—O4^iii^	2.3786 (12)	O1W—H1WB	0.832 (16)
Na1—O1	2.4016 (12)	O2W—H2WA	0.826 (16)
Na1—O1W	2.4017 (12)	O2W—H2WB	0.805 (16)
Na1—O2^iv^	2.4224 (13)		
			
O1W^i^—Zn1—O1W	180.00 (9)	O1—Na1—O2W^v^	92.61 (4)
O1W^i^—Zn1—O3	91.46 (4)	O1W—Na1—O2W^v^	90.58 (4)
O1W—Zn1—O3	88.54 (4)	O2^iv^—Na1—O2W^v^	74.93 (4)
O1W^i^—Zn1—O3^i^	88.54 (4)	O2—S1—O3	110.89 (7)
O1W—Zn1—O3^i^	91.46 (4)	O2—S1—O1	109.97 (6)
O3—Zn1—O3^i^	180.00 (8)	O3—S1—O1	110.00 (7)
O1W^i^—Zn1—O2W	92.62 (5)	O2—S1—O4	110.71 (7)
O1W—Zn1—O2W	87.38 (5)	O3—S1—O4	107.38 (6)
O3—Zn1—O2W	88.72 (4)	O1—S1—O4	107.81 (7)
O3^i^—Zn1—O2W	91.28 (4)	S1—O1—Na1	128.83 (7)
O1W^i^—Zn1—O2W^i^	87.38 (5)	S1—O2—Na1^vii^	117.79 (6)
O1W—Zn1—O2W^i^	92.62 (5)	S1—O2—Na1^v^	135.07 (7)
O3—Zn1—O2W^i^	91.28 (4)	Na1^vii^—O2—Na1^v^	103.29 (4)
O3^i^—Zn1—O2W^i^	88.72 (4)	S1—O3—Zn1	136.09 (7)
O2W—Zn1—O2W^i^	180.00 (7)	S1—O4—Na1^viii^	136.15 (7)
O2^ii^—Na1—O4^iii^	89.56 (4)	Zn1—O1W—Na1	126.34 (5)
O2^ii^—Na1—O1	112.94 (4)	Zn1—O1W—H1WA	113 (2)
O4^iii^—Na1—O1	105.09 (4)	Na1—O1W—H1WA	99.6 (19)
O2^ii^—Na1—O1W	155.87 (5)	Zn1—O1W—H1WB	107.7 (17)
O4^iii^—Na1—O1W	99.32 (5)	Na1—O1W—H1WB	102.0 (17)
O1—Na1—O1W	86.50 (4)	H1WA—O1W—H1WB	106 (2)
O2^ii^—Na1—O2^iv^	76.71 (4)	Zn1—O2W—Na1^iv^	113.77 (5)
O4^iii^—Na1—O2^iv^	89.57 (4)	Zn1—O2W—H2WA	117.7 (16)
O1—Na1—O2^iv^	162.11 (5)	Na1^iv^—O2W—H2WA	112.8 (17)
O1W—Na1—O2^iv^	80.94 (4)	Zn1—O2W—H2WB	114.1 (18)
O2^ii^—Na1—O2W^v^	75.00 (4)	Na1^iv^—O2W—H2WB	89 (2)
O4^iii^—Na1—O2W^v^	160.12 (5)	H2WA—O2W—H2WB	106 (2)

Symmetry codes: (i) −*x*, −*y*, −*z*; (ii) *x*, −*y*+1/2, *z*+1/2; (iii) −*x*+1, *y*−1/2, −*z*+1/2; (iv) −*x*, *y*−1/2, −*z*+1/2; (v) −*x*, *y*+1/2, −*z*+1/2; (vi) −*x*, −*y*, −*z*+1; (vii) *x*, −*y*+1/2, *z*−1/2; (viii) −*x*+1, *y*+1/2, −*z*+1/2.

### Hydrogen-bond geometry (Å, °)

**Table:**

*D*—H···*A*	*D*—H	H···*A*	*D*···*A*	*D*—H···*A*
O1W—H1WA···O1^iv^	0.800 (17)	1.916 (17)	2.6977 (17)	165 (3)
O1W—H1WB···O4^ix^	0.832 (16)	1.901 (16)	2.7288 (17)	173 (2)
O2W—H2WA···O1^iii^	0.826 (16)	2.051 (18)	2.8468 (16)	162 (2)
O2W—H2WB···O4^x^	0.805 (16)	2.15 (2)	2.8779 (16)	151 (3)

Symmetry codes: (iv) −*x*, *y*−1/2, −*z*+1/2; (ix) *x*−1, *y*, *z*; (iii) −*x*+1, *y*−1/2, −*z*+1/2; (x) −*x*+1, −*y*, −*z*.
